# Usual Interstitial Pneumonia and Lung Cancer

**DOI:** 10.7759/cureus.97862

**Published:** 2025-11-26

**Authors:** Lamiyae Senhaji, Meryem Karhate, Abir Bouhamdi, Mounia Serraj, Mohamed ElBiaze, Mohammed Chakib Benjelloun, Badreddine Alami, Bouchra Amara

**Affiliations:** 1 Department of Pulmonology, Sidi Mohamed Ben Abdellah University, Hassan II University Hospital, Fez, MAR; 2 Department of Pneumology, Hassan II University Hospital, Fez, MAR; 3 Department of Pneumology, Sidi Mohamed Ben Abdellah University, Hassan II University Hospital, Fez, MAR; 4 Department of Pulmonology, Hassan II University Hospital, Fez, MAR; 5 Department of Radiology, Hassan II University Hospital, Fez, MAR

**Keywords:** lung cancer, management, prognosis, uip, usual interstitial pneumonia

## Abstract

Background

Lung cancer is the leading cause of cancer-related mortality worldwide, with a notable association between interstitial lung diseases, particularly usual interstitial pneumonia (UIP), and lung cancer. This study investigates the epidemiology, clinical characteristics, histological findings, and treatment implications of this relationship.

Methodology

A retrospective study was conducted at the Department of Pneumology of Hassan II University Hospital in Fez, Morocco, from January 2016 to September 2024. Patient records of those with established UIP/idiopathic pulmonary fibrosis who developed lung cancer during follow-up, as well as lung cancer patients discussed in thoracic oncology multidisciplinary meetings with UIP pattern identified on CT, were reviewed. Data on demographics, clinical presentation, radiological and histological findings, treatment, and outcomes were collected and analyzed using Epi Info version 7.2.6.0.

Results

In total, 10 male patients with UIP and lung cancer were included, with a mean age of 66.2 years. Cough 8 (80%) and dyspnea 7 (70%) were the most common symptoms. Radiologically, 7 (70%) had definite UIP, and tumors were predominantly located in the lower lobes. Histological analysis revealed an equal distribution of adenocarcinoma and squamous carcinoma at four (40%) each, with six (60%) patients diagnosed at stage IV. Treatment varied, with one patient undergoing surgery, while others received chemotherapy or radiotherapy. Unfortunately, four patients died, highlighting the aggressive nature of the disease.

Conclusions

The association between UIP and lung cancer presents significant clinical challenges, impacting treatment strategies and survival outcomes. Continued research is vital to developing effective management protocols. A multidisciplinary approach is essential to optimize care for patients facing the dual burden of UIP and lung cancer, ultimately aiming to improve their prognosis and quality of life.

## Introduction

A growing body of evidence has established a significant association between interstitial lung diseases (ILDs) and the development of lung cancer, with the strongest link observed in patients with a usual interstitial pneumonia (UIP) pattern [1]. This connection is of paramount importance, as lung cancer continues to be the primary driver of cancer-related mortality on a global scale. This study aims to describe the clinical, radiological, and pathological characteristics of patients with UIP who develop lung cancer.

## Materials and methods

A retrospective, observational study was conducted in the Department of Pneumology at Hassan II University Hospital in Fez, Morocco, from January 2016 to September 2024. The study population consisted of the following two groups: patients with established UIP/idiopathic pulmonary fibrosis (IPF) who were regularly followed in our department and subsequently developed lung cancer, and patients diagnosed with lung cancer discussed in the thoracic oncology multidisciplinary team meeting (MDT) in whom a UIP pattern was identified on chest CT during oncological workup.

This study was conducted in accordance with the principles of the Declaration of Helsinki. The study protocol was approved by the University Hospital Ethics Committee of Fez (approval number: 03/22). Due to the retrospective nature of the study, the requirement for informed consent was waived. All patient data were anonymized to ensure confidentiality.

**Figure 1:**
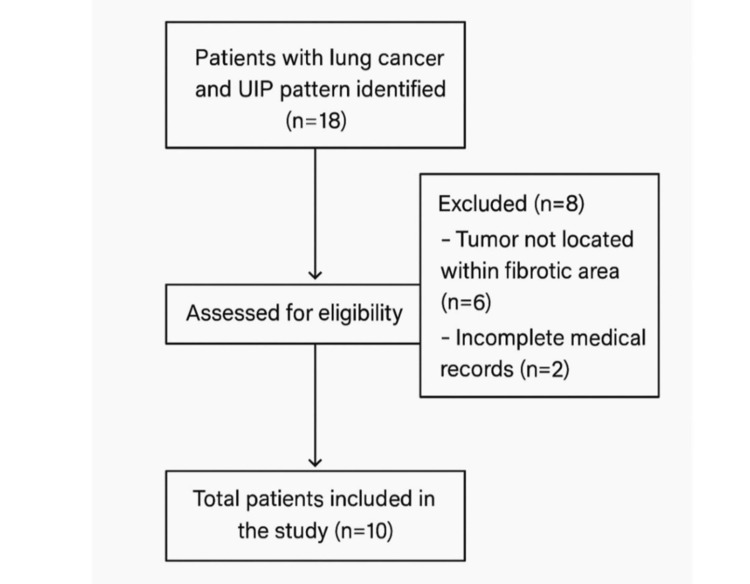
Flowchart illustrating the patient selection process.

The inclusion criteria for this study were as follows: (1) a radiological diagnosis of a UIP pattern on high-resolution CT (HRCT), according to the 2018 American Thoracic Society (ATS)/European Respiratory Society (ERS)/Japanese Respiratory Society (JRS)/Latin American Thoracic Association/Sociedad (ALAT) guidelines; (2) histologically or cytologically confirmed lung cancer; and (3) tumor location within or adjacent to fibrotic zones on imaging. Patients were excluded if (1) the tumor was located outside of the fibrotic areas; (2) the diagnosis of UIP or lung cancer could not be confirmed; or (3) medical records were incomplete (Figure 1).

The imaging protocol consisted of 5-mm thick slices from the apex to the base of the lungs. The radiological diagnosis of a UIP pattern was based on the 2018 ATS/ERS/JRS/ALAT guidelines and was confirmed by two experienced thoracic radiologists who reviewed the images independently.

No formal sample size calculation was performed for this study. Given the rarity of UIP/IPF as a clinical entity and the relatively low incidence of lung cancer in this population, patient recruitment was inherently limited. The final cohort of 10 patients represents the entire eligible population identified in our center over the eight-year study period. This limited sample size is acknowledged as a study limitation and precludes robust statistical analyses.

Data were extracted from electronic and paper medical records and included demographics (age, sex, smoking history, occupational exposure), clinical presentation, radiological data (HRCT patterns, tumor location and size, lymph node involvement), histological data (method of diagnosis, histological subtype), tumor staging according to the TNM classification (eighth edition), treatment modalities, and outcomes.

Descriptive statistics were used to summarize patient characteristics. Continuous variables were expressed as mean ± standard deviation or median with range, as appropriate. Categorical variables were presented as frequencies and percentages. Given the small sample size, no inferential statistical tests were performed. Data were documented in Microsoft Excel and analyzed using Epi Info version 7.2.6.0.

## Results

Patient characteristics

A total of 10 patients with UIP and lung cancer were enrolled in the study. All participants were male, with a mean age of 66.2 years (±6.49), ranging from 55 to 76 years. Among them, six (66.7%) were former smokers, two (22.2%) were current smokers, and one (11.1%) had never smoked. Additionally, two (22.2%) patients had occupational exposure (silica).

Clinical presentation

The most common symptom was cough, reported by eight (80%) participants, followed by dyspnea in seven (70%). Hemoptysis was noted in three (30%) patients. During the physical examination, five (55.5%) patients displayed finger clubbing as well as crackles, which were observed during pulmonary assessment.

Radiological and histopathological findings

**Figure 2:**
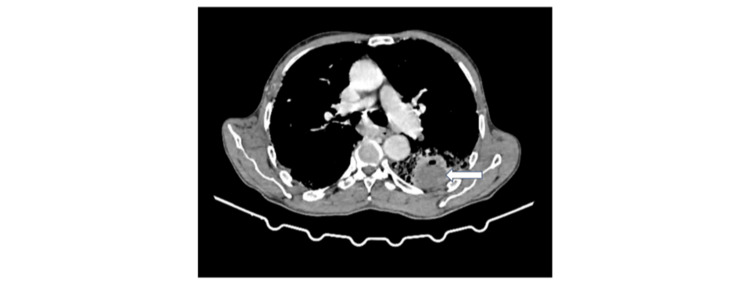
Cross-section of a thoracic CT scan: mediastinal window objectifying a left lower lobar tumor mass.

**Figure 3:**
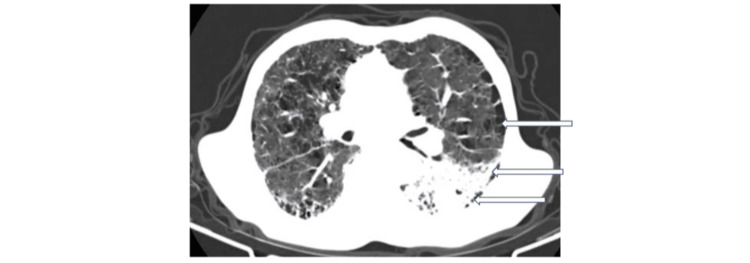
Cross-section of a thoracic CT scan: parenchymal window objectifying a left lower lobar tumor mass located within the area of fibrosis.

Radiological evaluations indicated that seven (70%) patients had definite UIP, while three (30%) had indeterminate UIP. In six (60%) cases, tumors were located in the lower lobes, and all tumors were situated within the fibrotic zones (Figures 2, 3).

The histological diagnosis was made through bronchoscopic endoscopy in six (60%) cases, a chest CT-guided biopsy of peripheral masses in three (30%) cases, and via pleural biopsy in one (10%) case with metastatic pleural effusion. The distribution of adenocarcinoma and squamous carcinoma was equal among the patients, each accounting for four (40%) patients. Tumor staging revealed that two (20%) were classified as stage IIIB, two (20%) as IIIC, and six (60%) as stage IV. Among the cohort, four (40%) patients had been monitored in our department for IPF before tumor diagnosis, while the UIP in other cases was discovered during the tumor evaluation. The average time between the IPF diagnosis and tumor detection was 27 months.

Treatment and outcomes

Therapeutic approaches demonstrated considerable variation, with only one (10%) patient receiving antifibrotic treatment due to financial constraints. Cancer-directed therapies included surgical intervention in one case, chemotherapy in four (44.5%) cases, and radiotherapy in two (22.2%) cases. Mortality analysis revealed four deaths, with three patients succumbing before treatment initiation and one experiencing acute respiratory failure following chemotherapy commencement. The average survival duration from tumor diagnosis to death was 27 days (Table 1).

**Table 1:** Baseline characteristics, clinical findings, and outcomes of patients (n = 10). HRCT = high-resolution CT; UIP = usual interstitial pneumonia

Characteristic	Value (n = 10)
Patient demographics	
Gender (male)	10 (100%)
Mean age (years, ±SD)	66.2 (±6.49)
Age range (years)	55–76
Smoking History	
Former smoker	6 (66.7%)
Current smoker	2 (22.2%)
Never smoked	1 (11.1%)
Occupational exposure	2 (22.2%)
Clinical presentation	
Symptoms	
Cough	8 (80%)
Dyspnea	7 (70%)
Hemoptysis	3 (30%)
Physical examination findings	
Finger clubbing	5 (55.5%)
Crackles	5 (55.5%)
Radiological findings	
UIP pattern on HRCT	
Definite UIP	7 (70%)
Indeterminate UIP	3 (30%)
Tumor location	
Lower lobes	6 (60%)
Histopathological findings	
Method of diagnosis	
Bronchoscopic endoscopy	6 (60%)
CT-guided biopsy	3 (30%)
Pleural biopsy	1 (10%)
Histological subtype	
Adenocarcinoma	4 (40%)
Squamous Carcinoma	4 (40%)
Tumor staging (TNM 8th edition)	
Stage IIIB	2 (20%)
Stage IIIC	2 (20%)
Stage IV	6 (60%)
Treatment and outcomes	
Antifibrotic treatment	1 (10%)
Cancer-directed therapy	
Surgery	1 (10%)
Chemotherapy	4 (44.5%)
Radiotherapy	2 (22.2%)
Mortality	4 (40%)
Mean survival (days)	27

## Discussion

The initial report linking lung carcinoma to interstitial pneumonia was made by Callahan et al. in 1952 [2]. However, their case involved acute interstitial pneumonia rather than the chronic form. Subsequently, in 1957, a study from Spain documented 12 cases of lung cancer occurring in patients with chronic interstitial pneumonia [1]. Meyer and Liebow later reported 36 instances of lung cancer associated with chronic interstitial pneumonia, providing detailed clinicopathological analyses [3].

Patients with idiopathic pulmonary fibrosis (IPF) are at significantly increased risk for developing lung cancer compared to the general population [4]. The incidence of lung cancer among IPF patients has been reported to range from 4.4% to 48% [5]. Notably, those with UIP have even higher rates, with up to 50% of UIP patients developing a concurrent malignancy [6]. This incidence starkly contrasts with a mere 9.1% in age-matched individuals without UIP [7]. Moreover, Yoo et al demonstrated that the cumulative risk for lung cancer in UIP patients escalates dramatically over time, with a 10-year risk of 31.1% [8].

In a study by Carobene et al., the median age at diagnosis of fibrosis and lung cancer was as follows: UIP/IPF-lung cancer, 820 months (68.3 years)/890 months (74.16 years). Male patients with active or previous smoking history were predominantly represented in all groups [9]. These results are close to ours.

Through detailed statistical examination, Turner-Warwick et al. first established the critical role of smoking habits as a risk factor for lung cancer among UIP patients [10]. Several studies confirmed that factors such as male gender, current smoking status, and a rapid decline in forced vital capacity also correlate with an increased risk of lung cancer in this population [8,11-13].

The pathophysiological link between UIP and lung cancer is multifaceted. The fibrotic environment of the lung, characterized by abnormal architectural changes, creates a conducive setting for oncogenesis. Studies indicate that lung cancer typically arises in fibrotic areas where normal lung tissue is replaced by destructive fibrosis. This relationship is likely mediated by the release of oncogenic factors, such as transforming growth factor-beta, which promotes fibroblast transformation, and genetic mutations, such as those in the *p53* gene [14,15]. Furthermore, abnormal activation of the Wnt/beta-catenin signaling pathway has been implicated in both conditions, contributing to resistance to apoptosis and fostering tumor growth within fibrotic tissues [16].

Diagnosing lung cancer in patients with UIP can be quite challenging. It is often hard to differentiate between diffuse inflammatory changes and malignant tumors. In the GERM“O”P study (Groupe d'Études et de Recherche sur les Maladies “Orphelines” Pulmonaires), histological diagnosis was impossible in nine (19%) patients [17]. HRCT is often the first step for diagnosis. It helps in identifying any suspicious nodules or masses. For nodules measuring 8 mm or more, a positron emission tomography-computed tomography scan is strongly recommended. If fludeoxyglucose uptake indicates tumor lesions, the authors advised starting with minimally invasive diagnostic methods such as endobronchial biopsy, endobronchial ultrasound-guided transbronchial needle biopsy for cases with pathological lymph nodes, or transthoracic needle biopsy for peripheral lesions with a risk of pneumothorax. In cases where less invasive methods fail to provide a diagnosis, a surgical biopsy may be necessary [18].

Given the vulnerability of UIP patients to developing lung cancer, the most effective diagnostic strategy for lung cancer must be thoroughly evaluated. Tzouvelekis et al. recently proposed a screening algorithm recommending annual HRCT for all IPF patients to detect lung cancer. However, the benefits and risks of this method have not been assessed in IPF patients, especially considering that 90% of positive CT results were ultimately deemed false positives in the National Lung Screening Trial [19].

Adenocarcinoma has emerged as the most prevalent histological subtype of lung cancer in patients with UIP, although squamous cell carcinoma was historically more common [9,14,20,21]. In a cohort study, adenocarcinomas accounted for 17 of 43 cases of lung cancer associated with UIP, often noted in fibrotic areas [22]. In our series, both adenocarcinoma and squamous cell carcinoma are prevalent.

Tumors are predominantly located in the lower lobes of the lungs, where fibrotic tissue has subverted and replaced normal lung architecture [21,23-25]. Emerging evidence suggests that lung cancer in IPF patients lacks disease homogeneity. These malignancies may originate in honeycombing, emphysematous upper lobes, or preserved lung tissue distant from fibrotic or emphysematous changes. We propose that tumor features are location-dependent, driven by different pathophysiological pathways. Moreover, the risk of postoperative acute exacerbation of IPF may vary according to the site of cancer development [26].

These tumors typically exhibit poorer prognostic features, including larger sizes and greater incidences of lymph node metastasis [27], as seen in our patients, who all had mediastinal lymph nodes and a large tumor. In the study by Carobene et al., within the UIP/IPF-lung cancer cohort, disease staging revealed four cases of stage I, two of stage II, five of stage III, and six of stage IV [9] disease, whereas stage IV was the most prevalent among our patients.

Managing lung cancer in the context of UIP presents unique challenges, primarily due to the risk of exacerbations during standard treatments. No recommendations in the guidelines are available. Surgery, radiotherapy, and chemotherapy may provoke acute exacerbations of pre-existing ILD, leading to increased morbidity and mortality [28,29].

Acute exacerbations of UIP can occur following various lung cancer treatments. For instance, one meta-analysis demonstrated that the overall incidence of acute exacerbations of UIP postoperatively was 14.6% across 10 studies involving 2,202 patients (95% confidence interval = 9.8-20.1) [30]. Sublobar resections (wedge or segmentectomy) have been shown to reduce the odds of postoperative acute exacerbations compared to lobectomies, with an odds ratio (OR) of 0.521. However, the extent of resection does not significantly influence overall survival in UIP patients, indicating that careful selection of surgical candidates is crucial [30].

Sato et al. [31,32] established a scoring system to predict postoperative acute exacerbation of IPF, which includes the following risk factors: history of IPF exacerbation, surgical approach, presence of UIP pattern, male gender, preoperative corticosteroid therapy, elevated Krebs von den Lungen-6 serum levels, and decreased %VC.

In a large cohort study, patients with a UIP pattern had a significantly higher incidence of chemotherapy-related exacerbation of ILD at 30% compared to just 8% in those without UIP. Moreover, the presence of UIP was associated with a higher risk of severe pulmonary toxicities during treatment, underscoring the need for careful monitoring and tailored treatment strategies [33]. That was the case of our patient who died after starting chemotherapy due to an acute exacerbation with severe pneumonia.

Chemotherapy regimens, particularly those involving drugs such as docetaxel, have been linked to exacerbations in patients with UIP [29]. A meta-analysis reported a significant risk of acute exacerbations related to ILD among patients treated with cytotoxic agents, highlighting the need for alternative approaches, such as the antifibrotic drugs pirfenidone and nintedanib. While nintedanib has shown promise in improving overall survival rates when combined with chemotherapy, its role in preventing acute exacerbations remains poorly defined [34].

Recent findings from the randomized phase III J-SONIC trial revealed significantly prolonged overall survival with the triple combination of nintedanib, carboplatin, and nanoparticle albumin-bound paclitaxel versus the doublet regimen of carboplatin and nab-paclitaxel in advanced non-squamous non-small-cell lung cancer (NSCLC) patients with concurrent IPF. Nevertheless, the observed incidence of acute IPF exacerbation was markedly lower than projected in both the chemotherapy and combination therapy cohorts [35]. The carboplatin plus nab-paclitaxel regimen confers reduced pneumonitis risk, as demonstrated by a 4.3% rate of acute ILD exacerbation reported in a multicenter phase II trial of NSCLC patients with baseline ILD [36].

The use of immune checkpoint inhibitors (ICIs) in lung cancer patients with pre-existing ILD is also contentious. Although some studies suggest favorable efficacy, the risk of immune-related pneumonitis is notably higher in this population. A careful assessment of risk-benefit ratios is essential when considering ICIs in patients with UIP [37].

Survival rates in lung cancer patients with UIP are generally poorer compared to those without UIP. For example, patients with a UIP pattern had a median overall survival of only 9.3 months, while those with a non-UIP pattern had a median overall survival of 16.5 months [38]. Another study indicated that survival at 36 months was 70% for patients without ILD, but only 30% for those with UIP [9]. Survival rates for patients with lung cancer and UIP are concerning, with a one-year survival rate of 60% [22]. This highlights the need for tailored treatment strategies that account for both the oncological and pulmonary aspects of patient care.

This study has several important limitations that must be acknowledged. First, its single-center, retrospective design and small sample size (n = 10) inherently limit the external validity and statistical power of our findings, precluding any robust subgroup analyses. Second, the absence of a control group, such as patients with UIP without lung cancer, or lung cancer without UIP, prevents us from drawing comparative conclusions or establishing a causal relationship between UIP and the observed outcomes. Third, a potential for selection bias exists, as our cohort was partly identified from patients discussed in MDT meetings. This approach may not capture all patients with UIP who develop lung cancer and could skew the sample toward more advanced or diagnostically challenging cases. Furthermore, the treatment protocols were not standardized, reflecting real-world clinical and financial variability within our setting. This heterogeneity in management makes it difficult to evaluate the efficacy of any specific therapeutic strategy. Finally, our outcome data is limited, particularly regarding long-term survival. The reported mean survival of 27 days reflects only the immediate, acute outcomes for a subset of deceased patients and should not be interpreted as a definitive prognostic indicator for this patient population as a whole. These limitations collectively underscore the exploratory and descriptive nature of our study. Despite these constraints, our findings provide a valuable preliminary snapshot of this patient population in our specific clinical setting and highlight the critical need for larger, multicenter prospective studies to better characterize this complex disease association.

## Conclusions

The association between UIP and lung cancer presents considerable clinical challenges, as illustrated by our single-center, descriptive study. The observations from our small cohort underscore the difficulties in managing these patients and are consistent with the poor prognosis often reported in the literature. Our findings highlight the importance of clinical vigilance in monitoring patients with UIP for the potential development of lung cancer. In our series, the interplay between fibrosis and malignancy appeared to complicate treatment decisions, with therapeutic options often limited by the risk of exacerbating the underlying lung disease. While the exploratory nature of this study precludes definitive conclusions on treatment strategies or survival impact, it does suggest that a multidisciplinary approach is essential for navigating the complex care of these patients. Further research, particularly through larger, multicenter registries and prospective studies, is needed to better understand this disease association and establish safer and more effective management protocols for this vulnerable population.
